# Commentary: “Mental Distress in Patients with Cerebral Visual Injury Assessed with the German Brief Symptom Inventory”

**DOI:** 10.3389/fnagi.2015.00122

**Published:** 2015-06-29

**Authors:** Ashley Craig

**Affiliations:** ^1^Kolling Institute of Medical Research, Sydney Medical School-Northern, Royal North Shore Hospital, The University of Sydney, St Leonards, NSW, Australia

**Keywords:** mental disorders, cerebral visual injury, adjustment to disease, mental distress, visual field defects

Visual disorders are associated with a substantial economic and personal burden (Rein, 2013). The recent paper by Gall et al. (2015) examined the influence of visual disorder on one aspect of this burden, mental health. Their study involved 122 participants (mean age = 58.1 ± 15.6 years, 72 males, 61% either married or in a relationship) who had visual field defects associated with cerebral visual injury due to either optic neuropathies or postchiasmatic lesions following ischemic or hemorrhagic stroke. About one-quarter of the participants also had additional impairments, such as hearing loss (*n* = 19), tactile paresthesia (*n* = 7), and loss of sense of smell (*n* = 5). Their paper highlights the importance of clarifying the mental health challenges associated with eye diseases, such as macular degeneration and diabetic retinopathy (Mathew et al., 2011). Conceivably, it would not be at all surprising if many people become highly distressed and depressive after receiving such a diagnosis. Concern and anticipation about one’s future health, social relationships, work status, and financial security would naturally increase, given expected increased difficulties with daily living and resultant deterioration in quality of life that such diseases can produce (Gall et al., 2015).

Gall et al. (2015) argue that the mental health impacts of cerebral visual injury, a result of optic neuropathies or lesions following ischemic or hemorrhagic stroke, is under investigated, and so requires clarification. To assess mental health, the investigators employed the brief symptom inventory (BSI), a well-validated psychometric tool that assesses domains, such as depressive mood, anxiety, hostility, and interpersonal sensitivity (Franke, 2015). Participants who were listed on a clinical database were invited to complete the BSI in addition to socio-demographic information. Cross-sectional mental health data on levels of psychological distress in the participants were compared to community norms for the BSI. Their findings indicated that cerebral visual injury is associated with elevated rates of mental health problems and concerns. For instance, they found over 25% of their sample reported clinical levels of psychological distress. While the cross-sectional nature of their study does not allow the conclusion of causal relationship, the most likely explanation is that the visual disorder leads to physical, social, behavioral, and personal anxieties and apprehensions, with the eventual possibility of leading to mental disorders like major depressive disorder. Similar dynamics have been shown to be associated with a number of physical disorders and diseases. For example, adults with a fluency disorder (chronic stuttering), a neurologically inherited disorder of communication, have been shown to be higher at risk of social anxiety disorder and elevated psychological distress including depressive mood and anxiety (Blumgart et al., 2010; Tran et al., 2011).

Furthermore, Gall et al. (2015) also found that participants with multisensory impairment (that is, visual impairment as well as impairment of other senses, such as smell and hearing) had significantly elevated levels of mental distress compared to those with visual impairment alone. This again is not surprising, and is not dissimilar to what we see with other physical disorders, like for instance, in people sustaining an acute spinal cord injury as well as a co-morbid traumatic brain injury (Craig et al., 2013, 2015). Gall et al. (2015) also suggested that quality of life will improve should mental health problems be addressed. This is certainly a positive way forward when dealing with impairment that is difficult to treat or perhaps not reversible. Arguably, assisting the person with the impairment to be more resilient by teaching skills designed to improve the way they cope and adjust with their disorder and impairment will prove to be a very attractive strategy. While the research findings of Gall et al. (2015) may seem evident, their data showing a strong connection between mental distress and a disorder like cerebral visual injury is highly important for directing future research in the area.

## Conflict of Interest Statement

The author declares that the research was conducted in the absence of any commercial or financial relationships that could be construed as a potential conflict of interest.
